# Reduced Exercise Tolerance and Pulmonary Capillary Recruitment with Remote Secondhand Smoke Exposure

**DOI:** 10.1371/journal.pone.0034393

**Published:** 2012-04-06

**Authors:** Mehrdad Arjomandi, Thaddeus Haight, Nasrat Sadeghi, Rita Redberg, Warren M. Gold

**Affiliations:** 1 University of California San Francisco Flight Attendants Medical Research Institute Center of Excellence, San Francisco, California, United States of America; 2 Cardiovascular Research Institute, University of California San Francisco, San Francisco, California, United States of America; 3 Department of Medicine, University of California San Francisco, San Francisco, California, United States of America; 4 San Francisco Veterans Affairs Medical Center, San Francisco, California, United States of America; 5 School of Public Health, University of California Berkeley, Berkeley, California, United States of America; 6 Radboud University Medical Center, Nijmegen, Netherlands; Idaho State University, United States of America

## Abstract

**Rationale:**

Flight attendants who worked on commercial aircraft before the smoking ban in flights (pre-ban FAs) were exposed to high levels of secondhand smoke (SHS). We previously showed never-smoking pre-ban FAs to have reduced diffusing capacity (Dco) at rest.

**Methods:**

To determine whether pre-ban FAs increase their Dco and pulmonary blood flow (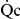) during exercise, we administered a symptom-limited supine-posture progressively increasing cycle exercise test to determine the maximum work (watts) and oxygen uptake (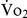) achieved by FAs. After 30 min rest, we then measured Dco and 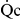 at 20, 40, 60, and 80 percent of maximum observed work.

**Results:**

The FAs with abnormal resting Dco achieved a lower level of maximum predicted work and 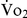 compared to those with normal resting Dco (mean±SEM; 88.7±2.9 vs. 102.5±3.1%predicted 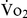; p = 0.001). Exercise limitation was associated with the FAs' FEV_1_ (r = 0.33; p = 0.003). The Dco increased less with exercise in those with abnormal resting Dco (mean±SEM: 1.36±0.16 vs. 1.90±0.16 ml/min/mmHg per 20% increase in predicted watts; p = 0.020), and amongst all FAs, the increase with exercise seemed to be incrementally lower in those with lower resting Dco. Exercise-induced increase in 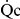 was not different in the two groups. However, the FAs with abnormal resting Dco had less augmentation of their Dco with increase in 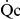 during exercise (mean±SEM: 0.93±0.06 vs. 1.47±0.09 ml/min/mmHg per L/min; p<0.0001). The Dco during exercise was inversely associated with years of exposure to SHS in those FAs with ≥10 years of pre-ban experience (r = −0.32; p = 0.032).

**Conclusions:**

This cohort of never-smoking FAs with SHS exposure showed exercise limitation based on their resting Dco. Those with lower resting Dco had reduced pulmonary capillary recruitment. Exposure to SHS in the aircraft cabin seemed to be a predictor for lower Dco during exercise.

## Introduction

Secondhand tobacco smoke (SHS) consists of the side-stream smoke from the burning end of the cigarette, which contains the highest concentration of particulate matter, and the exhaled mainstream smoke [1], [2]. Exposure to SHS is associated with diverse health effects in nonsmokers including heart disease, lung cancer, asthma flares, chronic obstructive pulmonary disease (COPD), and upper airway problems such as sinusitis [3], [4], [5], [6], [7], [8], [9], [10]. Occupational exposure to SHS presents a substantial health risk to workers [11], [12]. Flight attendants who worked on commercial aircraft before the ban on cigarette smoking (pre-ban FAs) experienced poor air quality and high levels of SHS in aircraft regardless of their class or cabin section [13], [14]. A pre-ban era chemical analysis of post-flight urine samples from these FAs showed elevated levels of urinary cotinine (a major metabolite of nicotine) close to levels currently observed in light or experimental smokers [15], [16], which signifies that the FAs had been exposed to substantial levels of tobacco smoke on these aircraft [17], [18].

We previously showed a cohort of healthy never-smoking pre-ban FAs with significant history of exposure to SHS had abnormal lung function [19]. This cohort had curvilinear flow-volume curves (concave to the volume axis) and reduced airflow at mid and low lung volumes. More impressively, over half of the cohort had abnormal single breath carbon monoxide diffusing capacity (DcoSB) below the lower 95% prediction limit based on Crapo's reference equations [20].

To further characterize the pulmonary function abnormalities in this cohort of pre-ban FAs, we performed 1-min interval, progressive incremental, symptom-limited cardiopulmonary exercise testing in the supine posture and then measured diffusing capacity at incremental workloads during exercise. Our hypothesis was that since cardiopulmonary exercise is a more sensitive tool for detecting lung function abnormalities, all of pre-ban FAs would have an abnormal exercise response. In particular, we hypothesized that the pre-ban FAs with abnormal resting DcoSB (lower than 95% prediction limit) would have lower pulmonary capillary recruitment with exercise compared to those with normal levels of resting DcoSB (above the lower 95% prediction limit), and that at least some of the FAs with normal levels of resting DcoSB would also show lower exercise-induced increase in their diffusing capacity, indicating that even the FAs with “normal” resting DcoSB might have reduced pulmonary capillary recruitment.

## Methods

### Ethics Statement

The UCSF Institutional Review Board (IRB), the Committee on Human Research, approved this study. Written IRB-approved informed consent was obtained from all study participants.

### Study Design

This was an observational cross-sectional study with convenience sampling of pre-ban FAs. Full details of the study methods are available in (Methods S1).

### Study Population

Between July 2003 and December 2010, we recruited pre-ban female FAs as part of a clinical investigation of the health effects of the cabin environment on flight attendants employed before and after the ban on smoking on commercial aircraft. Flight attendants were eligible to participate in the study if they had worked for at least five years on aircraft before the airline ban on cigarette smoking, were never-smokers (smoked less than 100 cigarettes lifetime), and had no previous clinical diagnosis of cardiac, pulmonary, or other diseases that could have adversely affected their pulmonary function. All subjects completed health and SHS exposure questionnaires [19], had a physical examination, and underwent pulmonary function testing and cardiopulmonary exercise testing. Full details of our methods are available in (Methods S1).

### Cardiopulmonary Exercise testing

Following an explanation of the exercise studies, the subject performed a physician-supervised, symptom-limited progressively increasing exercise test in the supine position on an electromagnetically braked, supine cycle ergometer (Medical Positioning Inc. Kansas City, MO). Subjects were advised to do their best, but otherwise were not encouraged; they could stop voluntarily at any time they believed they could not continue. We continuously monitored heart rate, blood pressure (BP), electrocardiogram (ECG), and breath-by-breath gas exchange.

The protocol consisted of 3-min rest, 1-min unloaded (freewheeling) cycling at 60 rpm, followed by increasing work rate of 20–30 Watts to a maximum tolerated, and 5-min of recovery. Twelve lead ECGs were monitored continuously and were recorded along with BP every 2 min. Oxyhemoglobin saturation (O_2_sat) determined by pulse oximetry was recorded continuously.

Subjects were rested for 30 min and a repeat exercise study was performed. In this second exercise study, incremental exercise in the supine posture on the same supine cycle ergometer was conducted using 6-min stages at 20, 40, 60, and 80% of maximum observed work, measuring within breath diffusing capacity (DcoWB), pulmonary blood flow (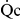), and HR in duplicate at each stage. Measurements of DcoWB and 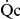 were performed using a rapid infrared analyzer system via breath-by-breath metabolic measurement as described extensively previously [21], [22], [23], [24], [25].

### Data Management and Analysis

Distributions of subjects' characteristics (i.e., age, pulmonary function) were computed for all subjects. Measures of pulmonary function at rest as well cardiac and respiratory responses to exercise, based on percent predicted of normal, were calculated and examined. Differences in characteristics, pulmonary function, and exercise responses between the two groups of FAs with resting DcoSB below or above the lower 95% prediction limit were examined using Student's t-test.

The percent predicted values for DcoWB at 40% maximum observed exercise were calculated using reference equations from Huang et al measured at 40% maximum observed exercise (DcoWB at rest = −0.057*age+0.221*height-11.525; DcoWB at 40% exercise = −0.023*age+0.324*height-25.273; reference equations for women) [22], and Charloux et al (DcoWB = 1.77*
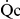
+12.16; reference equation not stratified by gender) [26].

Generalized estimating equations were used to compute regression lines for changes in DcoWB and 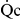 with increasing exercise as well as changes in DcoWB with 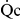. The differences in exercise-induced changes in DcoWb and 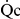 between the groups of FAs were examined using an interaction term in the regression models.

Linear regression models were used to examine the association between resting DcoSB, DcoWB at baseline (0% work), and DcoWB at 40% maximum observed work and years of aircraft cabin SHS exposure. To account for other potential cabin factors [13], [27], the years of SHS exposure (pre-ban years of employment) was adjusted for total years of employment in regression models. The association between lung function and years of cabin SHS exposure were modeled using LOWESS smoother (locally weighted scatterplot smoothing) analysis. Based on these models, a subgroup of pre-ban FAs with history of more than 10 years of cabin SHS exposure was identified and used for further analysis of the associations. All analyses were conducted in STATA (version 12.0).

## Results

### Subjects characteristics

Characteristics of the pre-ban FAs are shown in Table 1. Overall, 80 FAs were recruited for the study. Subjects were all healthy women between the ages of 41 and 76 who were never-smokers as defined by a lifetime history of less than 100 cigarettes use. None of the subjects were obese as defined by a body mass index (BMI)≥30, and none of them had any history of cardiopulmonary diseases or systemic diseases that may affect the cardiopulmonary function. All subjects exercised regularly (3 to 5 times weekly).

**Table 1:** Characteristics of pre-ban flight attendants.

Subject Characteristics	All FAs	FA with normal Dco	FA with abnormal Dco	p-value
Number	80	40	40	-
Age (years)	60.3±6.7	61.2±6.0	59.4±7.3	0.231
Height (cm)	164±5	164±6	165±5	0.321
BMI (kg/m^2^)	23.7±3.1	23.6±2.8	23.8±3.3	0.794
Hemoglobin level (g/dl)	14.1±1.1	14.2±1.1	13.9±1.1	0.213
DcoSB adjusted for Hgb (ml/min/mmHg)	20.0±3.0	21.7±2.9	18.3±2.1	**<0.0001**
DcoSB adjusted for Hgb (% predicted)	77.0±1.1	84.6±7.6	69.5±5.1	**<0.0001**
DcoSB adjusted for Hgb & alveolar volume (ml/min/mmHg)	4.3±0.5	4.4±0.5	4.1±0.5	**0.004**
DcoSB adjusted for Hgb & alveolar volume (% predicted)	81.3±9.5	84.9±9.2	77.8±8.6	**0.0006**
FEV_1_ (L)	2.56±0.38	2.60±0.31	2.51±0.43	0.289
FEV_1_ (% predicted)	102.5±13.7	105.8±13.8	99.3±12.6	**0.032**
FVC (L)	3.38±0.52	3.46±0.48	3.30±0.55	0.152
FVC (% predicted)	106.8±14.2	110.4±14.1	103.3±13.6	**0.023**
FEV_1_/FVC	0.76±0.04	0.75±0.04	0.76±0.05	0.635
TLC (L)	5.14±0.65	5.23±0.66	5.05±0.63	0.270
V_A_ (L)	4.70±0.61	4.91±0.53	4.49±0.61	**0.001**
TLC (% predicted)	100.5±10.0	102.3±9.5	98.6±10.3	0.141
V_A_ (% predicted)	91.4±10.3	95.8±8.7	87.1±10.1	**0.0001**
Air trapping [TLC – V_A_] (L)	0.46±0.34	0.34±0.39	0.58±0.24	**0.004**
Total length of employment (years)	26.8±10.0	26.5±10.6	27.1±9.5	0.840
Pre-ban length of employment (years)	18.4±8.2	18.4±7.6	18.5±8.8	0.940

Data is shown in mean ± standard deviation. Subjects were all female. Abbreviations: BMI: body mass index; DcoSB: single breath diffusing capacity; Hgb: hemoglobin; FEV_1_: forced expiratory volume in 1 second; FVC: forced vital capacity; TLC: total lung capacity measured by body plethysmography; V_A_: alveolar volume measured by single breath helium dilution.

Total years of airline employment varied in length between 6 and 40. The FAs' estimated length of exposure to aircraft cabin SHS (pre-smoking ban employment) was between 3 and 33 years representing a range of 15% to 100% of the total length of their active duty employment.

As we had shown previously in a smaller cohort [28], half of the subjects had DcoSB at rest below the 95% prediction limit of their normal values for their sex, age, and height, based on Crapo's reference equations [20] (40 FAs had “abnormal Dco” and 40 had “normal” Dco). While all FAs had normal FEV_1_, FVC, and FEV_1_ to FVC ratio (FEV_1_/FVC), those with abnormal resting DcoSB had a slightly lower FEV_1_ and FVC but similar FEV_1_/FVC (Table 1).

### Exercise Capacity

**Figure 1:**
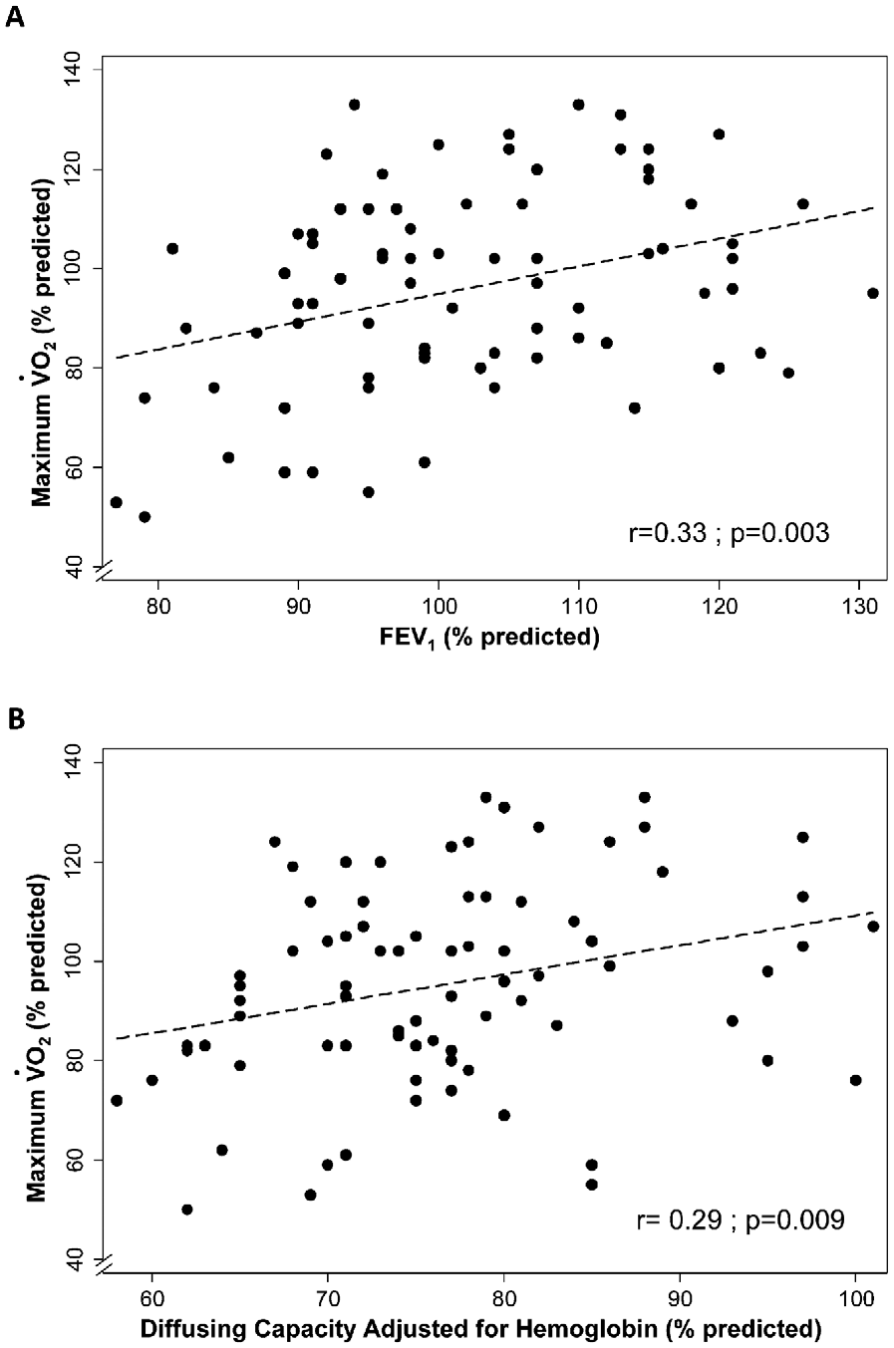
Association between exercise capacity and airflow and diffusing capacity in pre-ban FAs. The exercise capacity as estimated by maximum oxygen uptake (VO_2_) was directly associated with (**A**) FEV_1_ (r = 0.33; p = 0.002) and with (**B**) diffusing capacity at rest (r = 0.29; p = 0.008); r: correlation coefficient.

All FAs achieved a normal maximum exercise level based on maximum work and oxygen uptake (greater than 83% predicted value by Wasserman et al. [29]). However, the FAs with abnormal resting DcoSB achieved lower levels of maximum exercise as indicated by lower maximum predicted work rate (mean±SD: 123.9±31.6 vs. 132.6±24.2%predicted watts, p = 0.020), and lower maximum oxygen uptake (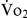
max) (1.24±0.30 vs. 1.39±0.26 L/min, p = 0.019 and 88.7±2.9 vs. 102.5±3.1%predicted 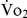
max, p = 0.001) (Table 2 & Figure S1). In regression models, the 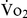
max was linearly associated with resting DcoSB (parameter estimate (PE)±SEM: 0.59±0.22; r = 0.29; p = 0.009) (Figure 1A) and with FEV_1_ (PE±SEM: 0.54±0.18; r = 0.33; p = 0.003) (Figure 1B) across all subjects. The Borg score for shortness of breath fatigue, and effort at maximum observed work [30], [31] was not significantly different between the two groups of FAs.

**Table 2:** Exercise Capacity.

Subject Characteristics	All FAs	FA with normal Dco	FA with abnormal Dco	p-value
Maximum work achieved (watts)	128.3±28.3	132.6±24.2	123.9±31.6	0.169
Maximum work achieved (% predicted watts)	128.9±33.2	137.4±30.2	120.3±34.3	**0.020**
Maximum 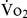 (L/min)	1.32±0.29	1.39±0.26	1.24±0.30	**0.019**
Maximum 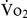 (% predicted 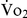 )	95.6±20.2	102.5±19.7	88.7±18.6	**0.001**
Maximum 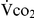 (L/min)	1.60±0.42	1.67±0.38	1.52±0.45	0.154
R at maximum work	1.23±0.14	1.23±0.12	1.24±0.16	0.706
Shortness of Breath (Borg Scale)	4.2±2.0	4.5±2.1	3.8±1.9	0.158
Effort (Borg Scale)	5.0±1.9	5.2±2.0	4.7±1.8	0.278
Fatigue (Borg Scale)	4.6±2.0	4.9±2.1	4.1±1.8	0.103

Data is shown in mean ± standard deviation. N = 80; abbreviations: 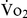: Oxygen uptake; 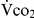: carbon dioxide output; R: respiratory gas exchange ratio.

### Cardiac Response to Exercise

The reduction in exercise capacity was associated with a significantly decreased maximum O_2_ pulse (oxygen uptake per heart beat) and decreased anaerobic threshold (AT) in FAs with lower resting DcoSB (Table S1). The stroke volume increased as expected with exercise in all FAs from a baseline of 0.067±0.022 (L/beat) measured in the supine posture at rest to 0.078±0.021 (L/beat) at 20% maximum observed exercise (p<0.0001), and then plateaued with increasing levels of exercise. The stroke volume and its pattern of change with exercise was not significantly different between the two groups of FAs, which suggests that the decreased aerobic capacity observed in pre-ban FAs with lower resting DcoSB was due to a smaller arteriovenous (A-V) oxygen difference in this group [29]. There was no difference in other cardiovascular measurements (Table S1).

### Respiratory Response to Exercise

The respiratory response to exercise in both groups was normal as reflected by the maximum levels of minute ventilation, respiratory gas exchange ratio (R), respiratory rate, tidal volume, total inspiratory time as a fraction of total respiratory cycle (Ti/Tot), and ventilatory equivalent of CO_2_ (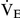/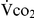) (Table S2).

### Diffusing Capacity during Exercise

The mean absolute difference between DcoWB and DcoSB values measured at rest in all FAs was 1.54±1.96 ml/min/mmHg (p<0.0001). There was a significant correlation between the DcoWB and DcoSB values measured at rest in all FAs (Pearson's correlation coefficient = 0.67, p<0.0001) (Figure S2).

**Figure 2:**
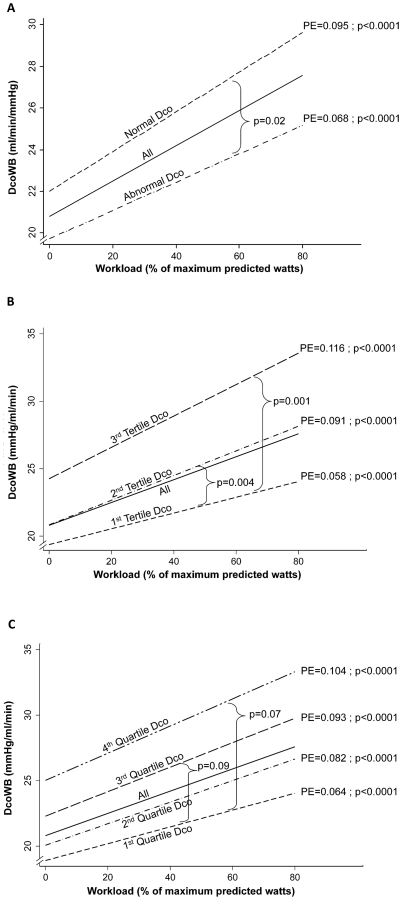
Association between within breath diffusing capacity (DcoWB) and workload. Generalized estimating equations were used to create linear regressions representing each association. Exercise-induced increase in diffusing capacity is decreased in flight attendants with lower diffusing capacity at rest. **A:** Stratification based on abnormal and normal diffusing capacity at rest. **B:** Stratification based on tertiles of diffusing capacity at rest. **C:** Stratification based on quartiles of diffusing capacity at rest. PE: Parameter estimate from regression models.

The DcoWB during exercise increased linearly with increasing work rate for all FAs. However the DcoWB in FAs with abnormal resting DcoSB increased less rapidly with increased work rate compared to the FAs with normal resting DcoSB (PE±SEM: 1.36±0.16 vs. 1.90±0.16 ml/min/mmHg per 20% increase in predicted watts; p = 0.020) (Figure 2A). The absolute and percent predicted (based on available reference equation measured at 40% maximum observed work [22]) differences between DcoWB values measured at 40% of maximum observed exercise between the two groups of FAs were 4.9±1.0 ml/min/mmHg (p<0.0001) and 13.8±3.0%predicted (p<0.0001), respectively (Table 3). Stratification of the FAs into tertiles or quartiles based on increasing resting DcoSB (15% or 10% increase in DcoSB, respectively) showed a similar pattern of lower increase in DcoWB with lower tertile or quartile of DcoSB (Figures 2B and 2C), suggesting that the effect of lower DcoSB on exercise-induced increase of DcoWB is incremental.

**Table 3:** Within-Breath Diffusing Capacity (DcoWB) at rest and with exercise.

Subject Characteristics	All FAs	FA with normal Dco	FA with abnormal Dco	p-value
DcoWB at rest (ml/min/mmHg)	20.1±3.5	21.4±3.6	18.7±2.8	**0.0004**
DcoWB at rest (% predicted Huang*)	93.8±14.9	100.8±14.7	86.5±11.2	**<0.0001**
DcoWB at rest (% predicted Charloux**)	96.3±21.4	103.8±20.3	88.7±20.0	**0.001**
DcoWB at 40% maximum observed work (ml/min/mmHg)	24.8±4.9	27.1±4.9	22.3±3.6	**<0.0001**
DcoWB at 40% maximum observed work (% predicted Huang*)	91.5±14.9	98.4±14.4	84.5±12.0	**<0.0001**
DcoWB at 40% maximum observed work (% predicted Charloux**)	91.7±18.0	98.8±18.2	84.0±14.3	**0.0002**

. Data is shown in mean ± standard deviation. Subjects were all female (N = 80).

* and ** : based on predicted values from Huang and Charloux, respectively [20], [24]. Abbreviation: DcoWB: within-breath diffusing capacity.

**Figure 3:**
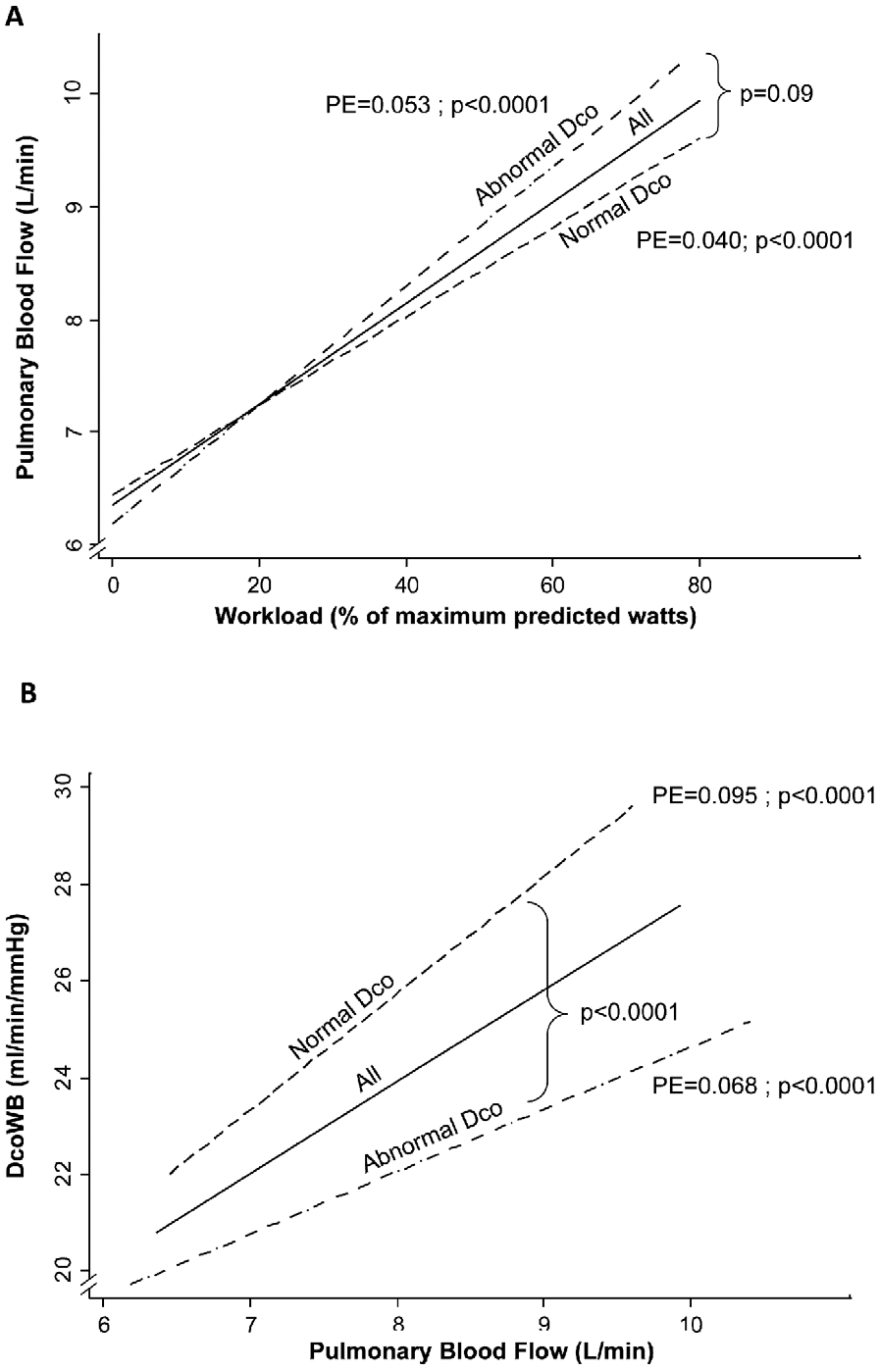
Association between within breath diffusing capacity (DcoWB), pulmonary blood flow, and workload. Generalized estimating equations were used to create linear regressions representing each association. **A:** Pulmonary blood flow increase with workload is not significantly different between the flight attendants with abnormal or normal diffusing capacity at rest. **B:** Diffusing capacity increases less with increasing blood flow in flight attendants with abnormal diffusing capacity at rest. PE: Parameter estimate from regression models.

The pulmonary flow (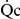) increased with increasing work rate in both groups of FAs and there were no significant differences in the rate of increase between the FAs with normal and abnormal resting DcoSB (PE±SEM: 0.053±0.007 vs. 0.040±0.004 L/min/%predicted watts; p = 0.097) (Figure 3A). However, the DcoWB increased less rapidly with increasing 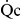 in FAs with abnormal resting DcoSB than in those with normal resting DcoSB: (PE±SEM: 0.093±0.06 vs. 1.47±0.09 ml/min/mmHg/L/min; p = 0.0001) (Figure 3B).

### Association with Exposure to SHS

**Figure 4:**
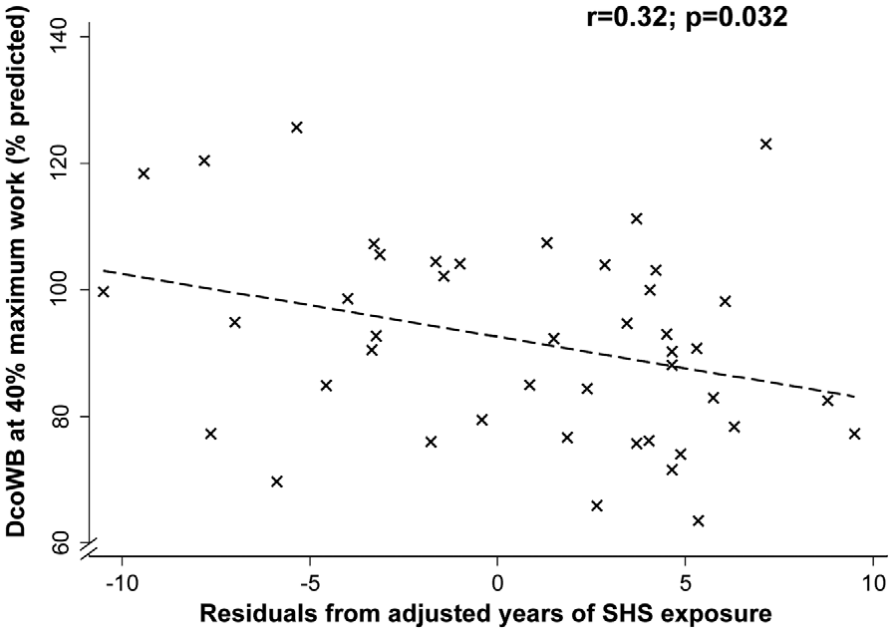
Association between within breath diffusing capacity (DcoWB) during exercise and years of cabin SHS exposure in flight attendants with ≥10 years pre-ban experience (N = 42). Percent predicted DcoWB at 40% maximum observed exercise was plotted against the residuals of adjusted pre-ban years employment. r: correlation coefficient.

The pre-ban length of employment (our proxy for aircraft cabin SHS exposure) was similar between the two groups of FAs with normal and abnormal DcoSB at rest. While neither DcoSB nor DcoWB at rest were associated with years of SHS exposure, DcoWb during exercise showed a trend towards inverse association with years of cabin SHS exposure. Using percent predicted equation by Huang et al [22], the pre-ban FAs, DcoWB at 40% maximum observed workload showed a trend inverse association with years of cabin SHS exposure (PE±SEM: −0.64%±0.37%, p = 0.095). In a subgroup of FAs with more than 10 years of cabin SHS exposure, the inverse association was statistically significant (PE±SEM: −0.99%±0.48%, r = 0.32 p = 0.032), suggesting a decrease of 0.99% in DcoWB for every year of SHS exposure beyond the first 10 years (Figure 4). Using percent prediction equations by Charloux et al [26] produced similar results (PE±SEM: −1.25%±0.55%; p = 0.029 at 40% workload and −1.19%±0.59%; p = 0.050 at 60% workload in the subgroup with history of more than 10 years of SHS exposure).

## Discussion

In this study of 80 never-smoking pre-ban FAs with significant history of exposure to SHS, we confirmed our previous findings in a smaller cohort that about half of the pre-ban FAs had decreased resting diffusing capacity (DcoSB) below the 95% prediction limit as well as curvilinear flow-volume curves (concave to the volume axis) and decreased air flow at mid and low lung volumes. More importantly, we found that those FAs with reduced resting DcoSB had lower exercise capacity as indicated by their maximum predicted work (watts) and maximum oxygen uptake (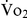). This lower exercise capacity was associated with decreased oxygen uptake per breath (O_2_ pulse) and decreased anaerobic threshold (anaerobic threshold was reached at lower absolute 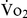 and lower % predicted value of maximum 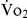). In addition, the exercise capacity was directly associated with their FEV_1_ at baseline. Furthermore, we found that while the two groups of FAs had similar pulmonary blood flow (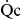), those with reduced DcoSB at rest increased their diffusing capacity with exercise (DcoWB) less with increasing workload and pulmonary blood flow, which indicates that despite exercise-induced increase in pulmonary blood flow, they had reduced pulmonary capillary recruitment. Finally, although no association between the resting diffusing capacity and SHS exposure was observed in our cohort, the diffusing capacity during exercise showed a trend association with the years of SHS exposure with the association reaching a significant level in those FAs with more than 10 years of exposure.

Our finding of decreased exercise capacity in the group of FAs with reduced resting diffusing capacity is interesting as none of FAs had any history of cardiopulmonary diseases and all reported exercising regularly 3 to 5 times weekly. Although the average maximum watts and average peak 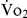 achieved by all the FAs were within the normal reference range, the FAs with reduced resting diffusing capacity achieved significantly lower levels compared to those with higher resting diffusing capacity, and many within the former group achieved maximum work levels well below the normal reference range (25% only achieved levels below the 77% maximum predicted 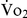). The lower exercise capacity of this group of FAs is likely due to decreased aerobic capacity as reflected by decreased 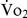 at AT and decreased O_2_ pulse. Since the stroke volume was similar between the two groups of FAs, the decreased aerobic capacity thus appears to be due to decreased arteriovenous (A-V) oxygen difference, which in turn suggests an abnormal distribution of oxygenated blood to non-essential tissues and reduced distribution of oxygenated blood to exercising muscle [29]. We also demonstrated that FEV_1_ was a predictor of exercise capacity in a linear fashion. The correlation coefficient of 0.33 between exercise capacity and FEV_1_ reflects a medium effect size according to the criteria of Cohen [32], and thus is consistent with a physiologically meaningful association. Given the presence of curvilinearity in flow-volume loops and decreased flow at mid and low lung volumes in the cohort, this association suggests that dynamic airflow obstruction may play a role in decreased exercise capacity of the FAs.

The decreased exercise capacity and lower exercise-induced increase in diffusing capacity in the FAs with reduced resting diffusing capacity further substantiate our previous findings of abnormal resting pulmonary function tests (i.e. decreased flow in low- and mid-lung volumes, air trapping, and reduced resting diffusing capacity) in this cohort with significant history of SHS exposure. The lower increase in exercise diffusing capacity, its incremental nature based on resting diffusing capacity, and lower ratio of diffusing capacity to pulmonary blood flow during exercise suggest that these FAs have reduced pulmonary capillary bed recruitment. Further anatomic or physiologic studies such as CT imaging or assessment of the inspiratory flow-volume loop assessment during exercise could help elucidate the underlying cause.

The finding that the FAs with reduced resting diffusing capacity had a lesser exercise-induced increase in their diffusing capacity with various stratification confirms our hypothesis that even some of the FAs with resting diffusing capacity within the 95% “normal” prediction limit had reduced pulmonary capillary recruitment. In addition, it shows that the diffusing capacity during exercise is a more sensitive measure of underlying abnormalities in pulmonary capillary bed than resting diffusing capacity. Together, these results show that the observation of a normal diffusing capacity at rest may not indicate that the pulmonary capillary recruitment during exercise will be normal.

The association between diffusing capacity during exercise and cabin years of SHS exposure had a two-tailed p-value of 0.095; however, if we assume that the adverse event on lung function would only be seen with increased SHS exposure (a one-tail assumption), the association then becomes significant (one-tail p-value of 0.048). In addition, the LOWESS smoother analysis showed a significant lung function and SHS exposure association (two-tailed p-value of 0.032) in the subgroup of FAs who had a longer SHS exposure history, a finding that is supported by the plausible biologic mechanism that higher exposure could cause higher adverse events. The correlation coefficient (r) of 0.32 between diffusing capacity during exercise and cabin years of SHS exposure represents a medium effect size according to the criteria of Cohen [32], and thus is not only statistically significant, but also is consistent with a meaningful association. Although most FAs did report additional SHS exposure apart from cabin exposure, it is expected that their non-cabin related SHS exposure was relatively insignificant compared to the intensity of their exposure while aboard aircraft [17]. In a previous study of this cohort, we investigated potential contribution of non-cabin related SHS exposure to subjects' lung function via comparison of the subjects with only cabin SHS exposure and those reported additional non-airline SHS exposure (i.e. exposure during childhood, adulthood, and/or non-airline employments), and did not find any significant differences in lung function between the two groups [19], which also suggest that non-cabin SHS exposure in FAs was dwarfed by their cabin SHS exposure.

Overall, our findings provide strong physiologic evidence consistent with presence of emphysema and/or COPD in this cohort of never-smoking FAs who were exposed to high concentrations of SHS for extended periods of time in the aircraft cabin. It remains unclear whether the pulmonary function abnormalities seen in this cohort are stable or progressive.

Our study has several possible limitations. First, and from a technical standpoint, performing the within breath diffusing capacity (DcoWB) measurement during exercise requires excellent self-control of breathing by the subject to maintain a low and constant expiratory flow, even if the subject is able to see and monitor flow on a screen during the maneuver. The measurement becomes increasingly difficult to perform at high workloads. To improve distribution of blood flow and ventilation-perfusion matching, we elected to perform our studies in the supine posture [33]. We aimed to attain maximum recruitment of the pulmonary capillary bed and diffusing capacity at a lower work rate during supine exercise rather than in the erect posture, thus easing the demands on the subject to control breathing during the test maneuver. The FAs who participated in our study were able to perform the DcoWB technique reliably during exercise from rest to 60% maximum observed work rate, but few were able to reach 80% let alone near maximum work rates. However, as shown in the results section, DcoWB increased with workloads and 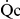 in a linear manner with no evidence of a plateau up to the maximum levels measured in FAs. Reproducibility of the DcoWB measurements was within 5%, which was considered satisfactory, and linear relationships were observed between DcoWB and 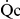 and were similar to those reported previously by other investigators [22], [26]. Second, in contrast to previous studies, we felt carbon monoxide (CO) backpressure on DcoWB measurement had to be considered. Stokes [34] and Huang [35] concluded the effect was negligible in their studies. Charloux et al found carboxyhemoglobin levels increased from 0.3% to 5.2% after 12 measurements in 4 subjects. In our study, carboxyhemoglobin levels increased from 0.3% to 10% after 12 measurements, and thus, we adjusted DcoWB for changes in hemoglobin and carboxyhemoglobin. Third, our study, which was a cross-sectional study of a group of FAs with a remote and unique high SHS exposure, did not include a similar control population for comparison. However, we used the available established prediction formulas to determine whether the obtained physiologic measurements were within the “normal” range. The appropriate control groups for our pre-ban FAs would be post-smoking ban flight attendants, who have been exposed to aircraft cabin [13], [27] except for SHS, and “ground-level” controls with no history of significant SHS exposure, both of which are challenging control groups as the post-ban flight attendants are in general younger and the “ground-level” controls do not have the specific exposure to non-SHS cabin factors that may contribute to lung function abnormalities. Of note, the normal predicted values include a wide range, and if one could show a longitudinal decline for an individual with time, even a value within the normal range may be considered to be abnormal. While our study was a cross-sectional one with measurements at a single time point, we were able to show that some FAs with normal resting diffusing capacity had abnormal physiologic responses to exercise, which suggest that despite having resting values within the “normal” range, they had abnormal pulmonary function. Fourth, the pulmonary function tests abnormalities of the FAs in this study do not meet the definition of COPD by GOLD criteria as the FAs had FEV_1_ within the normal predicted range and as they did not have any respiratory complaint at baseline. Traditionally, both COPD diagnosis and severity evaluation have been based on spirometry [36], [37], and change in FEV_1_ over time is still the most widely accepted measure of disease progression. However, FEV_1_ has limitations as it measures only one aspect of the disease and is not predictive of disease progression, especially in early disease [38], [39], [40]. In addition, COPD patients with similar FEV_1_ may show very different underlying pathologies, for example predominantly airspace disease (i.e. emphysema) or disease of the airways, as manifested by increased airway wall thickness [39]. Thus, we believe that despite the “normal” GOLD classification of the FAs in our cohort, the physiologic abnormalities that we have observed in them are best explained and most consistent with presence of COPD and emphysema [41]. Finally, we estimated the SHS exposure through the proxy of years of pre-ban employment using our occupational/employment questionnaire [28]. This method of SHS assessment may not be adequately sensitive for true amount of SHS exposure and may also be prone to recall bias. However, the cabin SHS exposure, which is relatively readily quantified using employment history, was much higher than levels experienced outside aircraft cabin in most circumstances [17], and dwarfs the non-cabin SHS exposure for these pre-ban FAs.

In conclusion, in this cohort of never-smoking pre-ban FAs with remote but significant history of cabin SHS exposure, we found physiologic abnormalities consistent with presence of emphysema and/or COPD. The FAs with lower diffusing capacity had exercise limitation and lower increase in their diffusing capacity with increasing workload and increasing pulmonary blood flow suggesting that those with lower resting diffusing capacity had reduced pulmonary capillary bed recruitment. Exposure to SHS in the aircraft cabin seemed to be a predictor for lower diffusing capacity during exercise in those with higher history of SHS exposure.

## Supporting Information

Figure S1
**Maximum work, maximum oxygen uptake (** 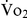 **), and maximum ventilatory equivalent of carbon dioxide (** 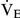 **/** 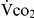 **) for never smoking pre-smoking ban flight attendants.** Black and white bars represent flight attendants with normal and abnormal resting diffusing capacity, respectively.(TIF)

Figure S2 **Correlation between single breath carbon monoxide diffusing capacity at rest (sitting position) and within breath diffusing capacity in supine position.**
(TIF)

Table S1
**Cardiovascular response to exercise.** Data is shown in mean ± standard deviation. * N = 80; subjects were all female. Abbreviations: SBP: systolic blood pressure; DBP: diastolic blood pressure; 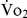: Oxygen uptake; AT; anaerobic threshold.(DOC)

Table S2
**Respiratory response to exercise.** Data is shown in mean ± standard deviation. * N = 80; subjects were all female. Abbreviations: SBP: systolic blood pressure; DBP: V_T_: tidal volume; diastolic blood pressure; 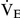: minute ventilation; 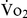: Oxygen uptake; 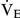/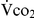: ventilatory equivalent of CO_2_; AT: anaerobic threshold; R: Respiratory gas exchange ratio.(DOC)

Methods S1 **Supplemental methods.**
(DOC)
