# Supplementary figures and images for: Reduced Exercise Tolerance and Pulmonary Capillary Recruitment with Remote Secondhand Smoke Exposure
Source: PLoS One. 2012 Apr 6;7(4):e34393. doi: 10.1371/journal.pone.0034393 (PMC3321018; doi:10.1371/journal.pone.0034393)

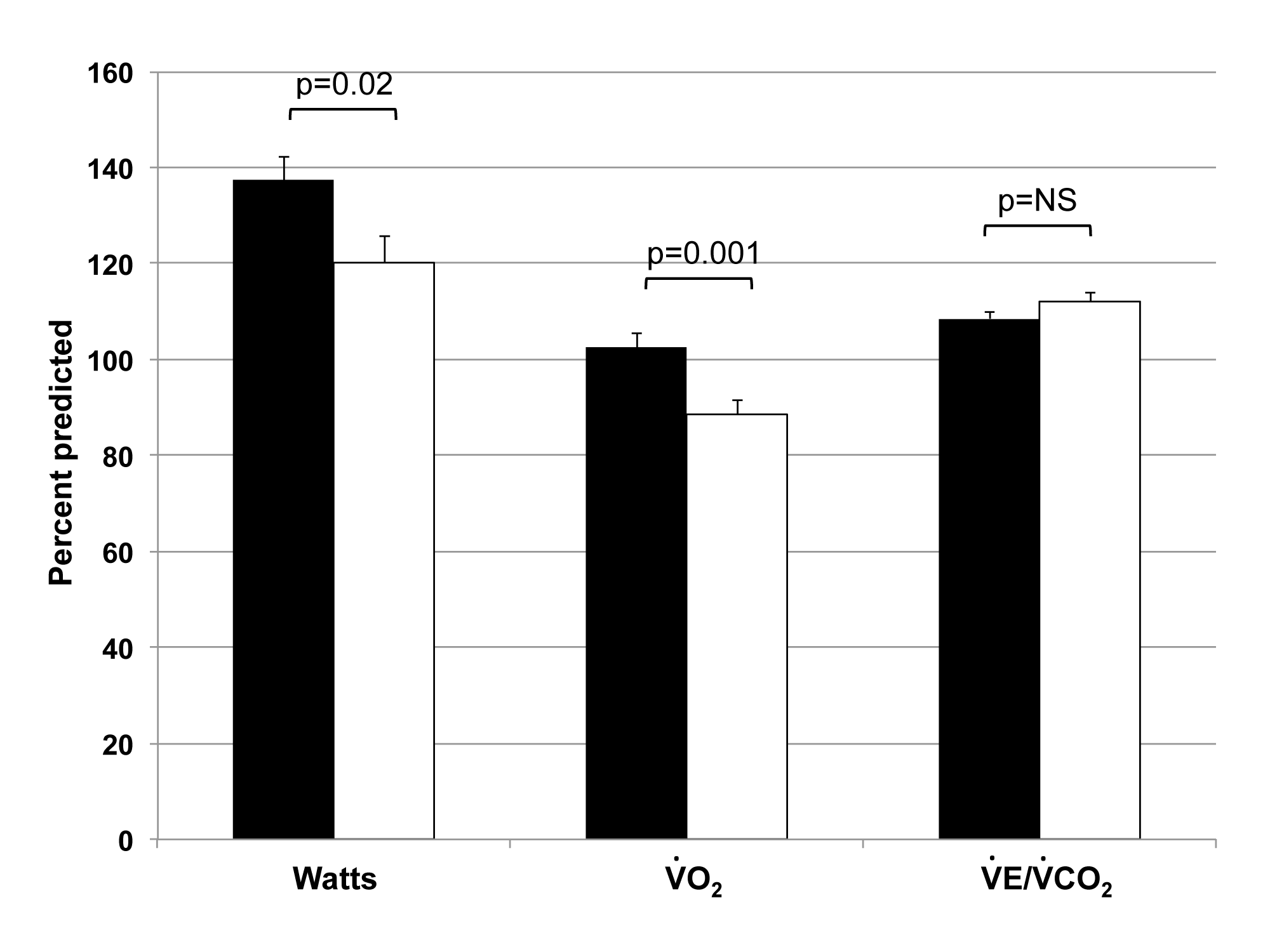

Supplement: Figure S1 — Maximum work, maximum oxygen uptake ( ), and maximum ventilatory equivalent of carbon dioxide ( / ) for never smoking pre-smoking ban flight attendants. Black and white bars represent flight attendants with normal and abnormal resting diffusing capacity, respectively. (TIF) [file pone.0034393.s001.tif]

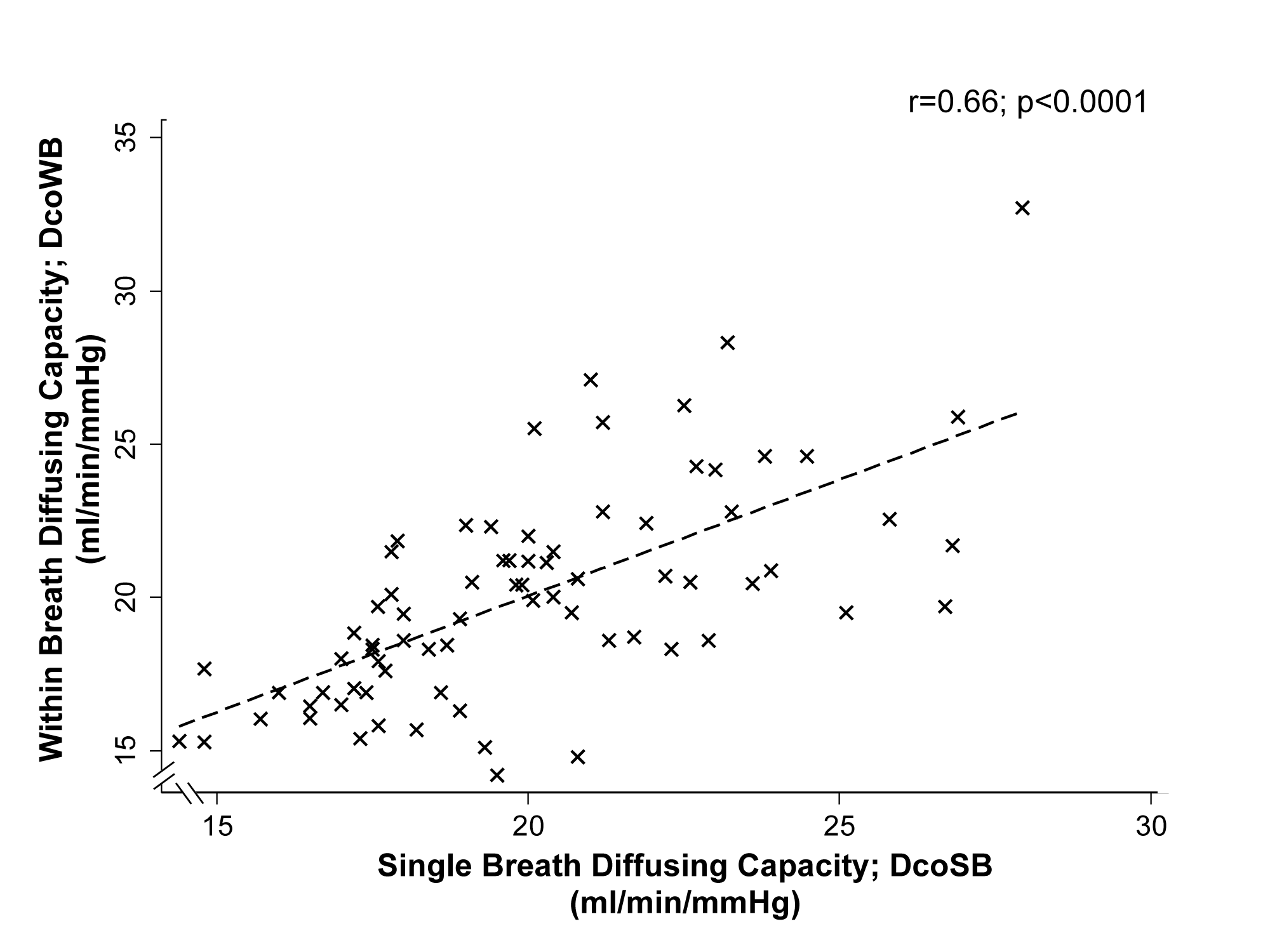

Supplement: Figure S2 — Correlation between single breath carbon monoxide diffusing capacity at rest (sitting position) and within breath diffusing capacity in supine position. (TIF) [file pone.0034393.s002.tif]
