# Supplementary material for: Reduced Exercise Tolerance and Pulmonary Capillary Recruitment with Remote Secondhand Smoke Exposure
Source: PLoS One. 2012 Apr 6;7(4):e34393. doi: 10.1371/journal.pone.0034393 (PMC3321018; doi:10.1371/journal.pone.0034393)
Supplement: Table S1 — Cardiovascular response to exercise. Data is shown in mean ± standard deviation. * N = 80; subjects were all female. Abbreviations: SBP: systolic blood pressure; DBP: diastolic blood pressure; : Oxygen uptake; AT; anaerobic threshold. (DOC) [file pone.0034393.s003.doc]

**Table S1-**

| **Subject Characteristics** | **All FAs** | **FA with normal Dco** | **FA with abnormal Dco** | **p-value** |
| --- | --- | --- | --- | --- |
| Maximum heart rate (% maximum predicted) | 87.2±12.6 | 87.9±14.1 | 86.5±11.0 | 0.623 |
| SBP at rest (mmHg) | 125±17 | 126±17 | 124±18 | 0.690 |
| SBP at maximum work  (mmHg) | 178±20 | 179±21 | 177±20 | 0.767 |
| DBP at rest (mmHg) | 75±9 | 75±9 | 75±9 | 0.927 |
| DBP at maximum work  (mmHg) | 90±11 | 92±10 | 90±12 | 0.487 |
| O_2_ pulse at maximum work (L/beat) | 9.56±1.94 | 10.22±1.89 | 8.91±1.79 | **0.002** |
| O_2_ pulse at maximum work (% predicted) | 112.2±23.8 | 120.7±23.0 | 103.6±21.6 | **0.001** |
| $\dot{\text{V}}\text{o}_{2}$ at AT (L/min) | 0.85±0.22 | 0.90±0.23 | 0.79±0.19 | **0.020** |
| $\dot{\text{V}}\text{o}_{2}$ at AT (% predicted maximum $\dot{\text{V}}\text{o}_{2}$) | 61.1±17.7 | 65.2±21.2 | 56.9±12.0 | **0.035** |
| $\dot{\text{V}}\text{o}_{2}$ at AT (% observed maximum $\dot{\text{V}}\text{o}_{2}$) | 65.2±14.2 | 65.4±13.0 | 65.0±15.4 | 0.899 |
| O_2_ pulse at AT (L/beat) | 7.86±2.48 | 8.18±2.44 | 7.55±2.51 | 0.262 |
| HR at AT (beats/min) | 110±24 | 110±29 | 111±19 | 0.882 |
