# Supplementary material for: Reduced Exercise Tolerance and Pulmonary Capillary Recruitment with Remote Secondhand Smoke Exposure
Source: PLoS One. 2012 Apr 6;7(4):e34393. doi: 10.1371/journal.pone.0034393 (PMC3321018; doi:10.1371/journal.pone.0034393)
Supplement: Table S2 — Respiratory response to exercise. Data is shown in mean ± standard deviation. * N = 80; subjects were all female. Abbreviations: SBP: systolic blood pressure; DBP: VT: tidal volume; diastolic blood pressure; : minute ventilation; : Oxygen uptake; /: ventilatory equivalent of CO2; AT: anaerobic threshold; R: Respiratory gas exchange ratio. (DOC) [file pone.0034393.s004.doc]

**Table S2-**

| **Subject Characteristics*** | **All FAs** | **FA with normal Dco** | **FA with abnormal Dco** | **p-value** |
| --- | --- | --- | --- | --- |
| RR (breath/min) | 35±9 | 35±8 | 35±10 | 0.912 |
| V_T_ at rest (L) | 0.62±0.17 | 0.60±0.15 | 0.65±0.19 | 0.228 |
| V_T_ at maximum work (L) | 1.52±0.32 | 1.58±0.34 | 1.46±0.30 | 0.103 |
| $\dot{\text{V}}_{\text{E}}$ at maximum work (% maximum predicted) | 51.6±12.4 | 52.0±12.1 | 51.2±12.9 | 0.768 |
| V_T_ at maximum work (% predicted) | 84.6±17.5 | 86.5±20.0 | 82.6±14.4 | 0.338 |
| Inspiratory time at maximum work (% of total respiratory cycle) | 49.0±2.9 | 49.2±2.8 | 48.7±3.0 | 0.545 |
| Maximum $\dot{\text{V}}_{\text{E}}$/$\dot{\text{V}}\text{co}_{2}$ | 32.2±3.4 | 31.9±2.4 | 32.6±4.2 | 0.316 |
| Maximum $\dot{\text{V}}_{\text{E}}$/$\dot{\text{V}}\text{co}_{2}$ (% predicted $\dot{\text{V}}_{\text{E}}$/$\dot{\text{V}}\text{co}_{2}$) | 110.2±11.0 | 108.5±8.2 | 112.0±13.2 | 0.162 |
| $\dot{\text{V}}_{\text{E}}$/$\dot{\text{V}}\text{co}_{2}$at AT | 33.8±3.6 | 32.6±3.8 | 33.8±3.4 | 0.151 |
| $\dot{\text{V}}_{\text{E}}$/$\dot{\text{V}}\text{co}_{2}$at AT (% observed maximum $\dot{\text{V}}_{\text{E}}$/$\dot{\text{V}}\text{co}_{2}$) | 103.5±11.9 | 104.4±11.2 | 102.5±12.5 | 0.112 |
| $\dot{\text{V}}_{\text{E}}$/$\dot{\text{V}}\text{co}_{2}$ at AT (% predicted maximum $\dot{\text{V}}_{\text{E}}$/$\dot{\text{V}}\text{co}_{2}$) | 112.1±17.5 | 111.0±12.1 | 113.1±21.7 | 0.593 |
| R at maximum work | 1.23±0.14 | 1.23±0.12 | 1.24±0.16 | 0.706 |
