# Supplementary material for: Reduced Exercise Tolerance and Pulmonary Capillary Recruitment with Remote Secondhand Smoke Exposure
Source: PLoS One. 2012 Apr 6;7(4):e34393. doi: 10.1371/journal.pone.0034393 (PMC3321018; doi:10.1371/journal.pone.0034393)
Supplement: Methods S1 — Supplemental methods. (DOC) [file pone.0034393.s005.doc]

**METHODS S1**

Study Design

This was an observational cross-sectional study with snowball sampling (convenience sampling in which recruitment is facilitated in an important way by “word-of-mouth” referral and information sharing) of pre-ban flight attendants.

Study Population

Between July 2003 and December 2010, we recruited pre-ban female flight attendants as part of a clinical investigation of the health effects of the cabin environment on flight attendants employed before and after the ban on smoking on commercial aircraft. Flight attendants were eligible to participate in the study if they had worked for at least five years on aircraft before the airline ban on cigarette smoking, were never-smokers (smoked less than 100 cigarettes lifetime), and had no previous clinical diagnosis of cardiac, pulmonary, or other diseases that could have adversely affected their pulmonary function. All subjects completed health and SHS exposure questionnaires, had a physical examination, and underwent pulmonary function testing and cardiopulmonary exercise testing.

From the 119 respondents, 21 subjects were excluded who were found to have history of tobacco use. Eleven other subjects were excluded due to history of illnesses that may have affected their lung function (Four subjects had history of breast cancer and radiation therapy, three had history of asthma, two had history of hepatitis C infection, one had received treatment for non-Hodgkin’s lymphoma, and one had history of sarcoidosis). Seven other subjects had BMI ≥ 30 and were also excluded. The remaining 80 subjects underwent pulmonary function and cardiopulmonary exercise testing.

Subjects received $50/hour for approximately a three-hour study period, plus a lunch voucher of $10 value, a beverage gift certificate of $5 value, and the cost of parking at the University of California San Francisco (USCF) Medical center. The UCSF Institutional Review Board, the Committee on Human Research, approved this study.

Health and SHS Exposure Characterization

Our SHS exposure questionnaire was developed based on a questionnaire previously used by UCSF FAMRI Center of Excellence investigators (1), and was modified to acquire information on airline-related occupational history including the employer airlines, the duration of employment, and the flight routes (domestic vs. international) as described previously (2). We defined “pre-ban years” as the number of years of employment before the smoking ban during which a flight attendant was exposed to SHS in the aircraft cabin, and used it as a quantitative proxy for exposure of flight attendants to SHS as described previously (2). To account for other potential cabin factors (3, 4), the pre-ban years of employment was adjusted for total years of employment in regression models examining the association between the results of pulmonary function tests and years of SHS exposure.

Pulmonary Function Testing

Routine pulmonary function tests were performed in the seated position using a model Vmax 229 CareFusion (CareFusion Corp., Yorba Linda, CA) and nSpire body plethysmograph (nSpire Health Inc., Longmont, CO) as described previously (2). This included measurement of flow-volume curve; spirometry (5); lung volume by single breath dilution (6, 7), and plethysmography (8); airway resistance during panting at functional residual capacity (FRC) (9, 10); and single breath carbon monoxide diffusing capacity (11). Pulmonary function studies were conducted according to the American Thoracic Society (ATS) and European Respiratory Society (ERS) guidelines (12-17). The largest coefficient of variation for diffusing capacity measurements in our laboratory among our biological standards is 4.1%.

Cardiopulmonary Exercise testing

Following an explanation of the exercise studies, the subject performed a physician-supervised, symptom-limited progressively increasing exercise test in the supine position on an electromagnetically braked, supine cycle ergometer (Medical Positioning Inc. Kansas City, MO). Subjects were advised to do their best, but otherwise were not encouraged; they could stop voluntarily at any time they believed they could not continue. We continuously monitored heart rate (HR), blood pressure (BP), electrocardiogram (ECG), and breath-by-breath gas exchange.

The protocol consisted of 3 min-rest, 1-min unloaded (freewheeling) cycling at 60 rpm, followed by increasing work rate of 20-30 Watts to a maximum tolerated, and 5-min of recovery. Twelve lead ECGs were monitored continuously. ECGs and BP, measured manually by a physician with a cuff, were recorded every 2 min. Oxyhemoglobin saturation (O_2_sat) determined by pulse oximetry was recorded continuously.

Minute ventilation (V_E_), oxygen uptake (VO_2_) and carbon dioxide output (VCO_2_) were measured breath-by-breath with an open-circuit metabolic cart (model Vmax 229, CareFusion, Yorba Linda, CA). The volumes of the flow meter, mouthpiece, and filter (70 mL x breathing frequency) were subtracted from V_E_ for the V_E_/VCO_2_ calculations. Anaerobic threshold (AT) was determined by the V-slope method (18, 19). Immediately before all tests, the gas analyzers was calibrated using reference gases of known concentrations and the ventilometer was calibrated using a 3-liter syringe (Hans Rudolph, Kansas MO). The metabolic system was verified using four trained technicians who provided monthly exercise values as biological standards for the Laboratory.

Subjects were rested for 30 min and a repeat exercise study was performed. In this second exercise study, incremental exercise in the supine posture on the same supine cycle ergometer was conducted using 6 min-stages at 20, 40, 60, and 80% of maximum observed work, measuring within breath diffusing capacity (DcoWB), pulmonary blood flow (Qc), and HR in duplicate at each stage.

Measurements of DcoWB and Qc were performed using a rapid infrared analyzer system via breath-by-breath metabolic measurement (model Vmax 229, CareFusion). The single exhalation within breath DcoWB and Qc methods and calculations have been described extensively previously (20-24). Rest and exercise cardiac output and diffusing capacity were assessed by a single slow exhalation of methane, acetylene, and carbon monoxide (21, 25, 26). Highly significant correlations have been reported between within breath Qc and Qc derived from radionuclide, thermodilution, and Fick methods (24, 27, 28). In brief, the subject performed a rapid inhalation from residual volume to total lung capacity of a test gas containing 0.3% CO, C_2_H_2_, and CH_4_. After a brief breath-hold (1-2 s), the subject exhaled slowly at a relative constant flow rate (0.5-1 L/s), which the subject was able to monitor on-screen. Gas concentrations were measured continuously during exhalation. A single exhalation DcoWB and single exhalation Qc were calculated by assessing the uptake of CO and C_2_H_2_ from the lung, with CH_4_ as an inert tracer gas, after washout of the dead space, and before closing volume (21, 27).

A blood sample (by finger-stick) was obtained at the end of the pulmonary function test for baseline hemoglobin and CO-hemoglobin and again at the end of the diffusing capacity-exercise for hemoglobin and CO-hemoglobin to appropriately adjust diffusing capacity measurements (15, 29).

Data Management and Analysis

Distributions of subjects’ characteristics (i.e., age, pulmonary function) were computed for all subjects. Measures of pulmonary function at rest as well cardiac and respiratory responses to exercise, based on percent predicted of normal, were calculated and examined. Differences in characteristics, pulmonary function, and exercise responses between the two groups of flight attendants with Dco at rest below or above the 95% prediction limit were examined using Student's t-test.

The percent predicted values for DcoWB at 40% maximum observed exercise were calculated using reference equations from Huang et al (DcoWB at rest = -0.057 * age + 0.221 * height - 11.525; DcoWB at 40% exercise = -0.023 * age + 0.324 * height - 25.273; reference equations for women) (21), and Charloux et al (DcoWB = 1.77 * Qc + 12.16; reference equation not stratified by gender) (26).

Generalized estimating equations were used to compute regression lines for changes in DcoWB and Qc with increasing exercise as well as changes in DcoWB with Qc. The differences in exercise-induced changes in DcoWb and Qc between the groups of flight attendants were examined using an interaction term in the regression models. Linear regression models were used to examine the association between resting DcoSB, resting DcoWB, and DcoWB at 40% maximum observed work and years of aircraft cabin SHS exposure. To account for other potential cabin factors (3, 4), the years of SHS exposure (pre-ban years of employment) was adjusted for total years of employment in regression models. All analyses were conducted in STATA (version 12.0).

**References:**

References

1. Eisner MD, Wang Y, Haight TJ, Balmes J, Hammond SK, Tager IB. Secondhand smoke exposure, pulmonary function, and cardiovascular mortality. *Ann Epidemiol* 2007;17:364-373.

2. Arjomandi M, Haight T, Redberg R, Gold WM. Pulmonary function abnormalities in never-smoking flight attendants exposed to secondhand tobacco smoke in the aircraft cabin. *J Occup Environ Med* 2009;51:639-646.

3. Lindgren T, Norback D, Andersson K, Dammstrom BG. Cabin environment and perception of cabin air quality among commercial aircrew. *Aviation, space, and environmental medicine* 2000;71:774-782.

4. Rayman RB. Cabin air quality: An overview. *Aviation, space, and environmental medicine* 2002;73:211-215.

5. Comroe Jr. JH. Pulmonary function tests. Methods in medical research. Chicago, Illinois: Year Book Publishers, Inc.; 1950. p. 188.

6. Mitchell MM, Renzetti AD, Jr. Evaluation of a single-breath method of measuring total lung capacity. *Am Rev Respir Dis* 1968;97:571-580.

7. Burns CB, Scheinhorn DJ. Evaluation of single-breath helium dilution total lung capacity in obstructive lung disease. *Am Rev Respir Dis* 1984;130:580-583.

8. Dubois AB, Botelho SY, Bedell GN, Marshall R, Comroe JH, Jr. A rapid plethysmographic method for measuring thoracic gas volume: A comparison with a nitrogen washout method for measuring functional residual capacity in normal subjects. *J Clin Invest* 1956;35:322-326.

9. Dubois AB, Botelho SY, Comroe JH, Jr. A new method for measuring airway resistance in man using a body plethysmograph: Values in normal subjects and in patients with respiratory disease. *J Clin Invest* 1956;35:327-335.

10. Briscoe WA, Dubois AB. The relationship between airway resistance, airway conductance and lung volume in subjects of different age and body size. *J Clin Invest* 1958;37:1279-1285.

11. Blakemore WS, Forster RE, Morton JW, Ogilvie CM. A standardized breath holding technique for the clinical measurement of the diffusing capacity of the lung for carbon monoxide. *J Clin Invest* 1957;36:1-17.

12. Miller MR, Crapo R, Hankinson J, Brusasco V, Burgos F, Casaburi R, Coates A, Enright P, van der Grinten CP, Gustafsson P, et al. General considerations for lung function testing. *Eur Respir J* 2005;26:153-161.

13. Miller MR, Hankinson J, Brusasco V, Burgos F, Casaburi R, Coates A, Crapo R, Enright P, van der Grinten CP, Gustafsson P, et al. Standardisation of spirometry. *Eur Respir J* 2005;26:319-338.

14. Wanger J, Clausen JL, Coates A, Pedersen OF, Brusasco V, Burgos F, Casaburi R, Crapo R, Enright P, van der Grinten CP, et al. Standardisation of the measurement of lung volumes. *Eur Respir J* 2005;26:511-522.

15. Macintyre N, Crapo RO, Viegi G, Johnson DC, van der Grinten CP, Brusasco V, Burgos F, Casaburi R, Coates A, Enright P, et al. Standardisation of the single-breath determination of carbon monoxide uptake in the lung. *Eur Respir J* 2005;26:720-735.

16. Pellegrino R, Viegi G, Brusasco V, Crapo RO, Burgos F, Casaburi R, Coates A, van der Grinten CP, Gustafsson P, Hankinson J, et al. Interpretative strategies for lung function tests. *Eur Respir J* 2005;26:948-968.

17. Standardization of spirometry, 1994 update. American thoracic society. *American journal of respiratory and critical care medicine* 1995;152:1107-1136.

18. Beaver WL, Wasserman K, Whipp BJ. A new method for detecting anaerobic threshold by gas exchange. *J Appl Physiol* 1986;60:2020-2027.

19. Wasserman K, Hansen JE, Sue DY, BJ W. Principles of exercise testing and interpretation. Philadelphia: Lea & Febiger; 1987.

20. Newth CJ, Cotton DJ, Nadel JA. Pulmonary diffusing capacity measured at multiple intervals during a single exhalation in man. *J Appl Physiol* 1977;43:617-625.

21. Huang YC, Helms MJ, MacIntyre NR. Normal values for single exhalation diffusing capacity and pulmonary capillary blood flow in sitting, supine positions, and during mild exercise. *Chest* 1994;105:501-508.

22. Martonen TB, Wilson AF. Theoretical basis of single breath gas absorption tests. *J Math Biol* 1982;14:203-220.

23. Wilson AF, Hearne J, Brenner M, Alfonso R. Measurement of transfer factor during constant exhalation. *Thorax* 1994;49:1121-1126.

24. Ramage JE, Jr., Coleman RE, MacIntyre NR. Rest and exercise cardiac output and diffusing capacity assessed by a single slow exhalation of methane, acetylene, and carbon monoxide. *Chest* 1987;92:44-50.

25. Stokes DL, MacIntyre NR, Nadel JA. Nonlinear increases in diffusing capacity during exercise by seated and supine subjects. *J Appl Physiol* 1981;51:858-863.

26. Charloux A, Enache I, Richard R, Oswald-Mammosser M, Lonsdorfer-Wolf E, Piquard F, Geny B. Diffusing capacity of the lung for co and pulmonary blood flow during incremental and intermittent exercise. *Scand J Med Sci Sports* 2009;20:e121-129.

27. Zenger MR, Brenner M, Haruno M, Mahon D, Wilson AF. Measurement of cardiac output by automated single-breath technique, and comparison with thermodilution and fick methods in patients with cardiac disease. *Am J Cardiol* 1993;71:105-109.

28. Elkayam U, Wilson AF, Morrison J, Meltzer P, Davis J, Klosterman P, Louvier J, Henry WL. Non-invasive measurement of cardiac output by a single breath constant expiratory technique. *Thorax* 1984;39:107-113.

29. Agostoni P, Wasserman K, Guazzi M, Cattadori G, Palermo P, Marenzi G, Guazzi MD. Exercise-induced hemoconcentration in heart failure due to dilated cardiomyopathy. *Am J Cardiol* 1999;83:278-280, A276.
